# Ambient ultraviolet radiation and ocular melanoma incidence in the United States, 2000−2019

**DOI:** 10.1038/s41433-024-02959-9

**Published:** 2024-02-13

**Authors:** Basilica M. Arockiaraj, Elizabeth K. Cahoon, Michael R. Sargen, Erping Long, Margaret A. Tucker, Jim Z. Mai

**Affiliations:** 1 Division of Cancer Epidemiology and Genetics, National Cancer Institute, National Institutes of Health, U.S. Department of Health and Human Services, Bethesda, MD USA; 2 School of Public Health, University of Maryland, College Park, MD USA; 3 Institute of Basic Medical Sciences, Chinese Academy of Medical Sciences and Peking Union Medical College, Beijing, China

**Keywords:** Risk factors, Eye cancer, Epidemiology

## Abstract

**Background/Objectives:**

Ocular melanoma is a rare, but deadly cancer. This large cancer registry study examines the associations between solar ultraviolet radiation (UVR) and incidence of different anatomical sites of ocular melanoma by sex, age, laterality, and race and ethnicity.

**Methods:**

Incidence data were derived from 21 cancer registries in the US for the years 2000–2019. Satellite-based UVR estimates were linked to county of residence at diagnosis. Incidence rate ratios (IRRs) and 95% confidence intervals (CIs) were calculated for UVR quartiles using Poisson models.

**Results:**

UVR was not associated with total ocular melanoma (*N* = 18,089) comparing Q4 versus Q1 (IRR = 0.98; 95%CI:0.94,1.03; *p-*trend = 0.07) or conjunctival melanoma (IRR = 0.99; 95%CI:0.82,1.19; *p*-trend = 0.81). However, in analyses of continuous UVR (per 10 mW/m^2^), risks were reduced for total ocular melanoma (IRR = 0.97; 95% CI: 0.96, 0.99). Incidence was increased for ciliary body/iris melanoma in the highest UVR quartile (IRR = 1.63; 95%CI:1.43,1.87; *p-*trend < 0.0001) and remained increased in non-Hispanic White individuals only. Incidence was reduced for choroidal melanoma in the highest UVR quartile (IRR = 0.86; 95%CI:0.82,0.91; *p*-trend < 0.0001).

**Conclusions:**

UVR may be associated with increased risk of ciliary body/iris melanoma. Reduced risk of choroidal melanoma may be due to higher diffuse UVR exposure to posterior ocular sites in locations at higher latitudes. Our results support and expand previous findings of associations of UVR using various surrogates on ocular melanoma risk and serve as a starting point for understanding the differences in the relationship between UVR and specific anatomical sites.

## Introduction

Ocular melanoma is diagnosed in about 2000 new patients in the United States each year [1]. About half of patients with uveal melanoma, the predominant anatomical site (~83%) of ocular melanoma, will develop metastases with a significant risk of both vision loss and death [2]. Mortality rates following metastases are 80% and 92% at 1 and 2 years, respectively [3]. Possible risk factors of ocular melanoma (mainly focused on uveal melanoma) include male sex, older age, light eye colour, sun-sensitive skin, higher numbers of cutaneous melanocytic nevi, ocular melanocytosis, ubiquitin carboxyl-terminal hydrolase *BAP1* germline mutations, and exposure to ultraviolet radiation (UVR) [4]. Treatment options for metastatic disease are limited [5], so identifying high risk groups and understanding risk factors of ocular melanoma is important for primary prevention and early detection. Recent whole-genome sequencing analyses found UV signature mutations in all eight patients with melanoma of the iris [6] and in both of two patients with conjunctival melanoma [7]. However, UV signature mutations can be induced by factors other than UVR exposure (e.g., reactive oxygen species) [8].

Although artificial UVR has been associated with increased risks of ocular melanoma, there is controversy in the association with solar UVR [4]. Epidemiological studies on the relationship between solar UVR and risk of ocular melanoma have been inconsistent [9–19]. Most previous studies examined surrogates of UVR such as place of birth [9, 11, 13, 14, 16], latitude [10, 17, 18], or were set in locations with low or having limited range of ambient UVR [10, 19]. Due to sample size limitations, few studies reported effect estimates across various anatomical sites of ocular melanoma, which may have differing exposures to ambient UVR, or factors such as race and ethnicity which may modify the relationship between UVR and ocular melanoma.

We examined the associations between ambient UVR and incidence of different anatomical sites of ocular melanoma by sex, age, laterality, and race and ethnicity using data from National Cancer Institute’s Surveillance, Epidemiology, and End Results (SEER) programme linked to United States county-level, satellite-based UVR. This is the largest study to examine association between ambient UVR and incidence of ocular melanoma, and the first study to examine such associations by anatomical sites and race and ethnicity using refined estimates of ambient UVR that have been used in epidemiological studies [20].

## Materials and methods

### Study population

Our study population included 21 population-based SEER cancer registries [21]. Counts of ocular melanoma were stratified by county of residence at diagnosis, sex, age at diagnosis (<50, 50–64, 65+ years), year of diagnosis (2000–2003, 2004–2007, 2008–2011, 2012–2015, 2016–2019), laterality (left-sided, right-sided, others), race and ethnicity (non-Hispanic White, Hispanic White, Black, Asian or Pacific Islander, and American Indian), and anatomical sites (conjunctiva, cornea, retina, choroid, ciliary body/iris, orbit not otherwise specified [NOS], overlapping lesion of eye and adnexa, and eye NOS). The county population counts used in the calculation of person-years at risk were based on the 2000 U.S. standard population (19 age groups—Census P25-1130). We excluded Alaska’s registry (N cases = 3) because Alaska was an outlier of UVR and only Alaskan Natives were included. Furthermore, we excluded cases with unknown age, race and ethnicity, or Federal Information Processing System code (*N* = 218).

### Ocular melanoma

We defined the first primary ocular melanoma cases as the international classification of disease for oncology coding of C69.0-C69.6 or C69.8-69.9 with histology code of 8720-8790. We excluded cases with unknown information on diagnostic confirmation (*N* = 397). Ocular melanoma cases included conjunctiva (C69.0), cornea (C69.1), retina (C69.2), choroid (C69.3), ciliary body/iris (C69.4), lacrimal gland (C69.5), orbit NOS (C69.6), overlapping region of eye and adnexa (C69.8), and eye NOS (C69.9). To accommodate potential differences in ascertainment of ocular melanoma diagnosis among registries, registry’s population size was created using census-based population size that was categorised into quartiles.

### Ambient UVR

Ambient UVR exposure was derived from the Total Ozone Mapping Spectrometer database [22]. Cloud-adjusted daily adjusted ambient UVB irradiance (305 nm wavelength) is provided on a 111 × 85 km (69 × 53 miles in the central United States) grid. Since the 1970’s, variation of UVR has been small besides the fluctuations of UVR during the eleven-year solar cycle. For the purposes of this study of ambient UVR and ocular melanoma, daily noontime estimates over 1982–1992 were averaged to represent the full solar cycle. The population centroid in 2000 of each SEER county was linked to the nearest yearly average UVR estimate. Counties within SEER were ranked by UVR and assigned quartiles Q1 to Q4 (lowest to highest).

### Statistical analysis

To examine the association between ambient UVR and ocular melanoma, we calculated incidence rate ratios (IRRs) and 95% confidence intervals (CIs) using Poisson regression adjusting for sex, age at diagnosis, race and ethnicity, year of diagnosis, and quartiles of registry’s population size. The natural logithm was applied to county-level populations as an offset in Poisson models. To assess dose-response, a test for trend was examined for a model that include ambient UVR quartile as an ordinal variable, and a model using continuous UVR per 10 mW/m^2^ (corresponding to about a 770-kilometer or 478 mile north-south distance on the east coast of the United States). To investigate whether the associations between continuous ambient UVR and incidence of ocular melanoma anatomical sites were modified by age, sex, and race and ethnicity, we included multiplicative interaction terms and conducted Wald tests in the Poisson models. Statistical tests were two-sided with a specified type I error of 0.05. Trend and interaction *P* values were corrected for multiple comparisons testing using Bonferroni adjustment. Sensitivity analyses were performed using a zero-inflated Poisson model, using different categories (terciles or quintiles) of ambient UVR, and all yielded similar results (data not shown). Due to limited number of cases in some anatomical sites, we included UVR analyses for total cases, sites with greater than 1,000 cases, and combined the other 6 sites. Poisson regression models were fitted with the GLIMMIX procedure using SAS 9.4 (SAS Institute Inc., Cary, NC, USA).

## Results

There were 18,089 ocular melanomas diagnosed between 2000 and 2019 (IR = 6.17/1,000,000 person-years; 95% CI: 6.08, 6.26) (Table 1). IRs of total cases were highest in older age groups, males, and non-Hispanic White. IRs of total cases were similar by year of diagnosis and laterality. These patterns of IRs for total cases by different factors were similar in the three major anatomical sites, including choroidal melanoma, ciliary body/iris melanoma and conjunctival melanoma (Table 1), and melanoma in other sites combined (Supplementary Table 1). Among tumours with available stage information, the proportions of regional or distant tumours were 7.6% for choroid 14.1% for ciliary body/iris, 24.1% for conjunctiva, and 30.3% for other sites (data not shown).

**Table 1:** Crude incidence rate for all ocular melanoma, three major anatomical sites with 1000+ cases and melanoma in other sites combined diagnosed by age, sex, race and ethnicity, diagnosis year, and laterality in the Surveillance, Epidemiology, and End Results Programme between 2000 and 2019.

	Total cases^a^		Choroid		Ciliary body/iris		Conjunctiva		Other sites^b^	
	Cases	IR (95% CI)	Cases	IR (95% CI)	Cases	IR (95% CI)	Cases	IR (95% CI)	Cases	IR (95% CI)
All	18,089	6.17 (6.08, 6.26)	13,812	4.71 (4.63, 4.79)	2013	0.69 (0.66, 0.72)	1079	0.37 (0.35, 0.39)	1185	0.40 (0.38, 0.43)
Age (years)										
<50	3393	1.66 (1.60, 1.72)	2528	1.24 (1.19, 1.29)	457	0.22 (0.20, 0.24)	215	0.11 (0.09, 0.12)	193	0.09 (0.08, 0.11)
50–64	6199	11.9 (11.7, 12.3)	4866	9.41 (9.15, 9.68)	663	1.28 (1.19, 1.38)	292	0.56 (0.50, 0.63)	378	0.73 (0.66, 0.81)
65+	8497	22.9 (22.4, 23.4)	6418	17.3 (16.9, 17.7)	893	2.41 (2.26, 2.58)	572	1.55 (1.42, 1.68)	614	1.66 (1.53, 1.80)
Sex										
Men	9370	6.49 (6.36, 6.62)	7192	4.98 (4.87, 5.10)	1024	0.71 (0.67, 0.75)	553	0.38 (0.35, 0.42)	601	0.42 (0.38, 0.45)
Women	8719	5.86 (5.74, 5.98)	6620	4.45 (4.34, 4.56)	989	0.66 (0.62, 0.71)	526	0.35 (0.32, 0.38)	584	0.39 (0.36, 0.43)
Race and ethnicity										
Non-Hispanic White	16,645	10.1 (9.91, 10.2)	12,801	7.74 (7.60, 7.87)	1868	1.13 (1.08, 1.18)	908	0.55 (0.51, 0.59)	1068	0.65 (0.61, 0.69)
Hispanic White	989	1.61 (1.51, 1.71)	705	1.15 (1.06, 1.23)	107	0.17 (0.14, 0.21)	104	0.17 (0.14, 0.20)	73	0.12 (0.09, 0.15)
Black	185	0.49 (0.42, 0.56)	127	0.33 (0.28, 0.40)	<25	^	27	0.07 (0.05, 0.10)	<25	^
Asian/Pacific Islander	227	0.92 (0.81, 1.05)	149	0.61 (0.51, 0.71)	<25	^	31	0.13 (0.09, 0.18)	26	0.11 (0.07, 0.16)
American Indian	43	1.20 (0.87, 1.62)	30	0.84 (0.56, 1.19)	<25	^	<25	^	<25	^
Year of diagnosis										
2000–03	3236	5.91 (5.71, 6.12)	2364	4.32 (4.14, 4.49)	375	0.68 (0.62, 0.76)	189	0.35 (0.30, 0.40)	308	0.56 (0.50, 0.63)
2004–07	3610	6.38 (6.17, 6.59)	2667	4.71 (4.53, 4.89)	399	0.70 (0.64, 0.78)	245	0.43 (0.38, 0.49)	299	0.53 (0.47, 0.59)
2008–11	3415	5.81 (5.61, 6.01)	2552	4.34 (4.17, 4.51)	390	0.66 (0.60, 0.73)	225	0.38 (0.33, 0.44)	248	0.42 (0.37, 0.48)
2012–15	3900	6.42 (6.22, 6.62)	3087	5.08 (4.90, 5.26)	400	0.66 (0.60, 0.73)	218	0.36 (0.31, 0.41)	195	0.32 (0.28, 0.37)
2016–19	3928	6.31 (6.11, 6.51)	3142	5.04 (4.87, 5.22)	449	0.72 (0.66, 0.79)	202	0.32 (0.28, 0.37)	135	0.22 (0.18, 0.36)
Laterality										
Left-sided	8863	3.02 (2.96, 3.09)	6787	2.31 (2.26, 2.37)	1019	0.35 (0.33, 0.37)	529	0.18 (0.17, 0.2)	528	0.18 (0.17, 0.20)
Right-sided	8994	3.07 (3.00, 3.13)	6919	2.36 (2.30, 2.42)	970	0.33 (0.31, 0.35)	536	0.18 (0.17, 0.2)	569	0.19 (0.18, 0.21)
Others^c^	232	0.08 (0.07, 0.09)	106	0.04 (0.03, 0.04)	<25	^	<25	^	88	0.03 (0.02, 0.04)

^, IR and IRR not calculated for fewer than 25 cases.

*IR* crude incidence rate (per 1,000,000 person-years), *CI* confidence interval.

^a^Total ocular melanoma included anatomical sites of choroid, ciliary body/iris, conjunctiva, eye NOS, overlapping region of eye and adnexa, orbit NOS, retina, cornea, and lacrimal gland, which ordered by number of cases.

^b^Melanoma in other sites combined included anatomical sites of eye NOS, overlapping region of eye and adnexa, orbit NOS, retina, cornea, and lacrimal gland.

^c^Others included not a paired site; only one side or side unspecified; bilateral, single primary; paired site: midline tumour; paired site, but no information concerning laterality.

In the total population, no association was observed for the highest quartile of UVR and total ocular melanoma (UVR Q4 *versus* Q1 IRR = 0.98; 95% CI: 0.94, 1.03; *p*-trend = 0.07) or conjunctival melanoma (IRR = 0.99; 95% CI: 0.82, 1.19; *p*-trend = 0.81) (Table 2). For choroidal melanoma, there was an inverse association for UVR Q4 *versus* Q1 (IRR = 0.86; 95% CI: 0.82, 0.91; *p*-trend < 0.0001). Risks were increased for UVR Q4 *versus* Q1 for ciliary body/iris melanoma (IRR = 1.63; 95% CI: 1.43, 1.87; *p*-trend < 0.0001), and for other sites combined (IRR = 1.66; 95% CI: 1.40, 1.97; *p*-trend < 0.0001). Similar patterns were observed when restricting to non-Hispanic White individuals which included the vast majority of cases.

**Table 2:** Incidence rate ratio of quartile of ambient ultraviolet radiation (UVR) for ocular melanoma, three major anatomical sites with 1000+ cases and melanoma in other sites combined in the surveillance, epidemiology, and end results programme between 2000 and 2019.

	Total population^a^					Non-Hispanic White^d^			
	Quartile of ambient UVR	Number of cases	Adjusted IRR^b^	95% CI	*p* for trend^c^	Number of cases	Adjusted IRR^b^	95% CI	*p* for trend^c^
Total cases^e^	Q1	5816	ref.		0.07	5517	ref.		0.06
	Q2	5168	1.09	1.05, 1.14		4879	1.09	1.03, 1.13	
	Q3	3559	0.98	0.93, 1.03		3168	0.99	0.99, 1.09	
	Q4	3546	0.98	0.94, 1.03		3081	0.98	0.93, 1.02	
Anatomical sites									
Choroid	Q1	4580	ref.		**<0.0001**	4361	ref.		**<0.0001**
	Q2	4013	1.07	1.02, 1.11		3808	1.07	0.98, 1.08	
	Q3	2732	0.94	0.89, 0.99		2452	0.95	0.92, 1.02	
	Q4	2487	0.86	0.82, 0.91		2180	0.85	0.80, 0.90	
Ciliary body/iris	Q1	538	ref.		**<0.0001**	512	ref.		**<0.0001**
	Q2	587	1.39	1.23, 1.56		557	1.39	1.24, 1.64	
	Q3	382	1.19	1.02, 1.37		347	1.21	1.33, 1.81	
	Q4	506	1.63	1.43, 1.87		452	1.66	1.46, 1.95	
Conjunctiva	Q1	346	ref.		0.81	315	ref.		0.68
	Q2	289	1.02	0.87, 1.20		254	1.00	0.85, 1.19	
	Q3	225	0.97	0.80, 1.18		175	0.97	0.78, 1.19	
	Q4	219	0.99	0.82, 1.19		164	0.97	0.79, 1.19	
Other sites^f^	Q1	352	ref.		**<0.0001**	329	ref.		**<0.0001**
	Q2	279	1.06	0.90, 1.24		260	1.08	0.92, 1.28	
	Q3	220	1.17	0.96, 1.42		194	1.24	1.01, 1.52	
	Q4	334	1.66	1.40, 1.97		285	1.69	1.41, 2.03	

Cutoffs for ambient UVR quartile: 17.8–<26.4 (referent), 26.5–< 40.1, 40.1–<45.0 and 45.0–76.0 mW/m^2^.

^a^Total populations by quartiles UVR were 2427,929,673, 2049,231,696, 2160,918,462 and 2158,328,571, respectively.

^b^Adjusted for sex, age at diagnosis (<50, 50–64, 65+), race and ethnicity (non-Hispanic White, others [Hispanic White, Black, Asian Pacific American, American Indian]), year of diagnosis and registry’s population size that was categorised into quartiles using census-based population size to create four roughly equal categories (Q1: Iowa, Utah, Seattle (Puget Sound) Registry, Atlanta (Metropolitan) Registry, Rural and Greater Georgia Registries, San Francisco-Oakland SMSA Registry, San Jose-Monterey Registry, Los Angeles Registry, and Hawaii; Q2: Louisiana, Illinois, and Greater CA Registry; Q3: New Mexico, New York, Connecticut, and New Jersey; Q4: Texas, Idaho, Massachusetts, and Kentucky).

^c^Trend *P* values for solar UVR were based on trend tests using ordinal UVR quartiles (1 through 4) with bold indicating significant after Bonferroni correction (10 tests).

^d^Populations of non-Hispanic White individuals by quartiles UVR were 1668,263,682, 1375,566,774, 964,597,881, 955,688,856, respectively.

^e^Total cases included anatomical sites of choroid, ciliary body/iris, conjunctiva, eye NOS, overlapping region of eye and adnexa, orbit NOS, retina, cornea, and lacrimal gland, which ordered by number of cases.

^f^Melanoma in other sites combined included anatomical sites of eye NOS, overlapping region of eye and adnexa, orbit NOS, retina, cornea, and lacrimal gland.

**Fig. 1:**
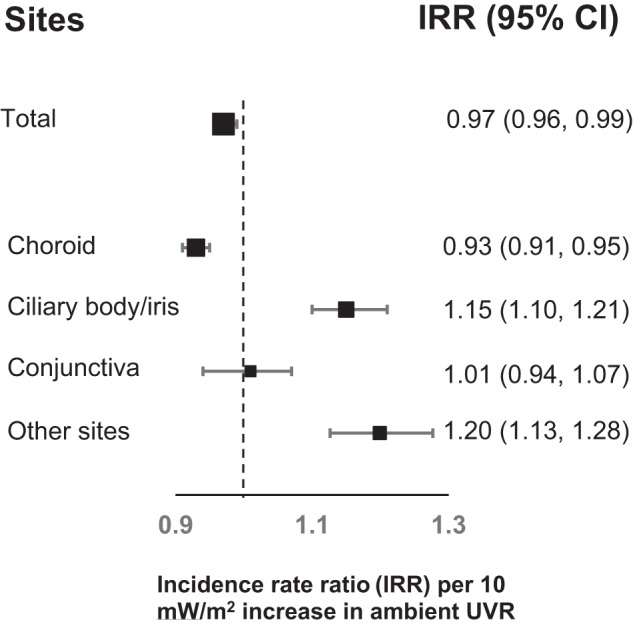
Risks of total cases of ocular melanomaa, choroidal melanoma, ciliary body/iris melanoma and conjunctival melanoma and melanoma in other sites combinedb associated with an increase of 10 mW/m^2^ in ambient UVR in the Surveillance, Epidemiology, and End Results Program, 2000–2019. ^a^Total ocular melanoma included anatomical sites of choroid, ciliary body/iris, conjunctiva, eye NOS, overlapping region of eye and adnexa, orbit NOS, retina, cornea, and lacrimal gland. ^b^Melanoma in other sites combined included anatomical sites of eye NOS, overlapping region of eye and adnexa, orbit NOS, retina, cornea, and lacrimal gland. Adjusted for age, sex, race and ethnicity, year of diagnosis, and registry’s population size that was categorised into quartiles using census-based population size to create four roughly equal categories (Q1: Iowa, Utah, Seattle (Puget Sound) Registry, Atlanta (Metropolitan) Registry, Rural and Greater Georgia Registries, San Francisco-Oakland SMSA Registry, San Jose-Monterey Registry, Los Angeles Registry, and Hawaii; Q2: Louisiana, Illinois, and Greater CA Registry; Q3: New Mexico, New York, Connecticut, and New Jersey; Q4: Texas, Idaho, Massachusetts, and Kentucky). Note: Square area is proportional to number of cases.10 mW/m^2^ corresponds to about a 770-kilometer (~478 mile) north-south distance on the East Coast of the United States.

In analyses of continuous UVR (per 10 mW/m^2^), risks were reduced for total ocular melanoma (IRR = 0.97; 95% CI: 0.96, 0.99) and choroidal melanoma (IRR = 0.93; 95% CI: 0.91, 0.95). Increased risks remained for ciliary body/iris melanoma (IRR = 1.15; 95% CI: 1.10, 1.21) and melanoma in other sites combined (IRR = 1.20; 95% CI: 1.13, 1.28) (Fig. 1).

The associations between continuous UVR and ocular melanoma incidence were not modified by age, sex, and race and ethnicity (*p*-interaction values were non-significant after Bonferroni correction; Table 3). The associations between continuous UVR and ocular melanoma incidence were similar and had overlapping CIs for the left- and right-sided.

**Table 3:** Incidence rate ratio of continuous UVR (per 10 mW/m^2^) for ocular melanoma, three major anatomical sites with 1000+ cases and melanoma in other sites combined by age, sex, race and ethnicity, and laterality in the Surveillance, Epidemiology, and End Results Programme between 2000 and 2019.

	Total cases^a^	Choroid	Ciliary body/iris	Conjunctiva	Other sites^c^
	IRR^b^ (95% CI)	IRR^b^ (95% CI)	IRR^b^ (95% CI)	IRR^b^ (95% CI)	IRR^b^ (95% CI)
Age (years)					
<50	0.99 (0.95, 1.03)	0.95 (0.91, 1.00)	1.17 (1.05, 1.30)	1.05 (0.90, 1.23)	1.04 (0.89, 1.22)
50–64	0.96 (0.94, 0.99)	0.92 (0.89, 0.95)	1.14 (1.05, 1.24)	1.02 (0.89, 1.15)	1.21 (1.09, 1.35)
65+	0.98 (0.95, 1.00)	0.93 (0.91, 0.96)	1.15 (1.07, 1.24)	0.99 (0.90, 1.08)	1.24 (1.14, 1.35)
Sex					
Men	0.97 (0.95, 0.99)	0.93 (0.90, 0.95)	1.16 (1.08, 1.24)	0.99 (0.91, 1.09)	1.20 (1.10, 1.31)
Women	0.98 (0.96, 1.00)	0.93 (0.91, 0.96)	1.14 (1.07, 1.23)	1.02 (0.92, 1.12)	1.20 (1.10, 1.31)
Race and ethnicity					
Non-Hispanic White	0.97 (0.96, 0.99)	0.93 (0.91, 0.95)	1.16 (1.10, 1.22)	0.99 (0.92, 1.07)	1.20 (1.12, 1.28)
Hispanic White	0.88 (0.81, 0.94)	0.85 (0.78, 0.93)	0.95 (0.76, 1.20)	0.92 (0.73, 1.16)	1.02 (0.76, 1.36)
Black	1.06 (0.87, 1.30)	1.14 (0.89, 1.45)	^	0.93 (0.54, 1.59)	^
Asian /Pacific Islander	1.00 (0.87, 1.15)	0.97 (0.81, 1.14)	^	1.00 (0.70, 1.42)	1.46 (1.01, 2.11)
American Indian	0.71 (0.53, 0.97)	0.61 (0.42, 0.88)	^	^	^
Laterality					
Left-sided	0.98 (0.96, 1.00)	0.94 (0.91, 0.96)	1.14 (1.07, 1.22)	0.99 (0.90, 1.09)	1.21 (1.10, 1.33)
Right-sided	0.97 (0.95, 0.99)	0.93 (0.91, 0.95)	1.15 (1.07, 1.23)	1.04 (0.94, 1.14)	1.15 (1.05, 1.25)
Others^d^	1.01 (0.87, 1.16)	0.67 (0.53, 0.83)	^	^	1.52 (1.21, 1.91)

^, IR and IRR not calculated for fewer than 25 cases.

Bonferroni-corrected *P* values for interaction for age, sex, and race and ethnicity were non-significant (15 tests). On the East Coast of the United States, 10 mW/m^2^ corresponds to about a 770-kilometer (~478 mile) north-south distance.

*IRR* incidence rate ratio, *CI* confidence interval.

^a^Total ocular melanoma included anatomical sites of choroid, ciliary body, conjunctiva, eye NOS, overlapping region of eye and adnexa, orbit NOS, retina, cornea, and lacrimal gland.

^b^Mutually adjusted for age, sex, and race and ethnicity race and ethnicity (non-Hispanic White, others [Hispanic White, Black, Asian Pacific American, American Indian]) as appropriate, in addition to year of diagnosis and registry’s population size that was categorised into quartiles using census-based population size to create four roughly equal categories (Q1: Iowa, Utah, Seattle (Puget Sound) Registry, Atlanta (Metropolitan) Registry, Rural and Greater Georgia Registries, San Francisco-Oakland SMSA Registry, San Jose-Monterey Registry, Los Angeles Registry, and Hawaii; Q2: Louisiana, Illinois, and Greater CA Registry; Q3: New Mexico, New York, Connecticut, and New Jersey; Q4: Texas, Idaho, Massachusetts, and Kentucky).

^c^Melanoma in other sites combined included anatomical sites of eye NOS, overlapping region of eye and adnexa, orbit NOS, retina, cornea, and lacrimal gland.

^d^Others included not a paired site; only one side or side unspecified; bilateral, single primary; paired site: midline tumour; paired site, but no information concerning laterality.

## Discussion

In this large U.S. cancer registry study with a broad range of ambient UVR exposure, we did not find an increased risk of ambient UVR with total ocular melanoma. However, ambient UVR was associated with increased risk of ciliary body/iris melanoma among non-Hispanic White individuals. We found some evidence of an association between ambient UVR and reduced risk of choroidal melanoma among non-Hispanic White and Hispanic White individuals.

### Total ocular melanoma

Previous studies of UVR and melanoma of the eye often reported all sites of ocular melanoma as a group [10, 14] or uveal melanoma (combinations of choroidal melanoma, ciliary body melanoma, and iris melanoma) (Supplementary Table 2) [9, 11–13, 15, 17–19]. Our null results for total ocular melanoma were consistent with two studies each that reported no relationships with latitude and modelled UVR exposure for all ocular melanoma [10, 14]. A number of studies of uveal melanoma also reported no relationship for surrogates of solar UVR exposure (e.g., modelled ambient UVR [11, 13], occupational sun exposure [15], sun exposure during vacation [12], eye protection [19], and others [9, 13]). However, other UVR surrogates did point to a suggestive positive association with risks of uveal melanoma for higher occupational sun exposure [19] and lack of eye protection [9, 13]. In addition, proximity to the Equator was associated with an increased risk in a case-control study of uveal melanoma (choroidal melanoma, ciliary body melanoma and iris melanoma) [9], but reduced risks were reported in another case-control study that did not include melanoma of the iris [13]. Although case-control studies usually collected detailed information on a number of UVR surrogates, they usually included small numbers of cases and were conducted in settings with a limited range of ambient UVR.

In contrast to case-control studies, cancer registry studies usually included a large number of cases with a relatively a broad range of ambient UVR, but little to no individual exposure data. Virgili et al. reported that decreasing latitude (i.e., higher exposure to solar UVR) was associated with reduced risks of choroidal melanomas, ciliary body/iris melanoma, and melanoma in the retina in Europe [18]. Yu et al. also showed reduced risks of uveal melanoma with decreasing latitude in 12 US cancer registries [17]. However, this study reported a suggestive increased risk of other types of ocular melanoma (conjunctival melanoma and melanoma in the cornea) [17].

As discussed previously, UVR may play different roles in different anatomical sites of ocular melanoma possibly because of the specific positions of the structures within the eye. It is generally believed that some UVR of wavelengths between 300 nm and 400 nm are able to reach the anterior uveal structures of the eye (shorter wavelengths are absorbed by the cornea), while only a very small amount of UVR reaches the posterior of the eye, primarily in older adulthood [23]. The conjunctiva and cornea are the most anterior segment of the eye, and the iris is the most anterior segment of the uveal tract. These anatomical sites have been suggested to almost completely absorb solar UVR. We found increased risks of melanoma in other sites combined. Other sites (*N* = 1185) largely represent C69.9 (eye NOS; *N* = 622) and C69.8 (overlapping region of eye and adnexa; *N* = 208), which are used when a single tumour overlaps an adjacent subsite or multiple tumours arise in different subsites, respectively. These sites are likely to represent cases with a later stage at diagnosis, so that interpreting UVR association is challenging as it may reflect cancer progression. Coding of some rare anatomical sites may reflect contiguous spread from adjacent melanocyte-containing tissues, though cases in which the site of origin is uncertain should be coded as C69.8 (overlapping region of eye and adnexa) or C69.9 (eye NOS) [24].

### UVR and increased risk of ciliary body/iris melanoma

We found a positive association for ambient UVR and ciliary body/iris melanoma, which included both ciliary body melanoma and iris melanoma. The iris may be the more UVR-exposed site as it is the most anterior segment of the uveal tract. Our results were consistent with a case-control study that found increased risk of iris melanoma in never use of eye protection in sun (*versus* almost always) [9]. Our findings were also supported by a whole-genome sequencing analysis of melanoma tumours representing all sites of the uveal tract, which found UV mutation signatures were restricted to those originating from the iris [6]. A challenge in interpreting this finding is that UV signature mutations may be acquired as the tumour evolves [25]. We also found positive association of ciliary body/iris melanoma in non-Hispanic White individuals only, which was similar to the known patterns by race and ethnicity in UVR-induced cutaneous melanoma. Our study may strengthen an etiological role of UVR in ciliary body/iris melanoma. However, this harmful association was not consistent with Vajdic et al. that found no association between several surrogates (e.g., latitude, occupational and recreational sun exposures, lifetime modelled ambient UVR, and eye protection) of UVR and risks of iris melanoma [16]. We observed similar incidence rates for ciliary body/iris melanoma across laterality (left versus right). Since automobile drivers in the United States would be more exposed on the left side, our findings do not support increased exposure to UVR from driving. It has been reported that more cutaneous melanomas occur on the left side of the body in the US [26], although a large multinational cancer registry study reported left-sided predominance even in countries/regions with right-sided driving patterns such as England, Scotland, and New South Wales, Australia [27].

### UVR and reduced risk of choroidal melanoma

Our findings of an inverse association for UVR and melanoma of the choroid were largely consistent with other cancer registry studies examining associations between latitude and risks of uveal melanoma, of which choroidal melanoma was predominant [17, 18]. Our findings were also somewhat consistent with a case-control study of choroidal melanoma and ciliary body melanoma combined among White individuals which reported an inverse association with ambient UVR, possibly because choroid comprised 91% of cases in the study [16].

Our finding of an inverse association between ambient UVR and choroidal melanoma was not consistent with some case-control studies using personal surrogates of UVR such as time outdoors, outdoor occupation and eye protection. For instance, Vajdic et al. showed both longer time outdoors on weekdays and total lifetime occupational hours of exposure were risk factors of choroidal melanoma and ciliary body melanoma combined in men, but not for other UVR surrogates examined in the study (e.g., recreational hours, lifetime ambient UVR, latitudes at birth or at diagnosis, and eye protection) [16].

We do not know the underlying mechanism for the inverse association of UVR in choroidal melanoma. A study examining the correlation between UVR dose distribution and tumour locations of choroid suggested that choroid was unlikely to be related to UVR because UVB was not transmitted through the lens and the cornea and did not reach the choroid. A very limited proportion of UVA was transmitted to the posterior pole, and even less to the anterior part of the choroid [28]. However, a more recent study supported a light-related aetiology for choroidal melanoma because tumour initiation was not uniformly distributed, with rates of occurrence concentrated in the macular area and decreasing progressively with increasing distance from the macular to the ciliary body [29]. One possibility is that our lower ambient UVR exposure at higher latitudes could result in higher UVR exposure to posterior ocular sites. Vajdic, Kricker, and Armstrong have highlighted that because of instinctive aversion responses to direct UVR and facial features and the anatomical configuration and geometry of the eye, the greatest exposure to the eye is from diffuse UVR [30]. Greater posterior ocular exposure to diffuse UVR occurs when the sun is near the horizon, and at higher latitudes, the sun is close to the horizon for a longer period each year than at lower latitudes [30]. Since our UVR measure is inversely correlated with latitude, our lower UVR estimates may be related to higher exposure to posterior ocular sites, which may result in the observed inverse association between ambient UVR and choroidal melanoma.

### No association between UVR and conjunctival melanoma

Our null association of conjunctival melanoma was consistent with Vajdic et al. that showed no strong evidence for sun exposure and melanomas of the conjunctiva [16]. However, the conjunctivas are on the front of the eye and are exposed to UVR directly. While a genome-wide sequencing study found UV mutation signature in conjunctival melanomas, such sequencing was conducted in only two non-Hispanic White patients [7].

Our null results for conjunctival melanoma was not consistent with Yu et al. who found marginally reduced risks of conjunctival melanoma and melanoma in the cornea combined for higher exposure to ambient UVR among non-Hispanic White individuals in US-based cancer registries [17]. Our study includes an additional 10 U.S. population-based cancer registries with a wider range of ambient UVR exposure and examines all races and ethnicities with conjunctival melanoma.

### Strengths and limitations

A major strength of this study is that the study population includes up to 48% of the U.S. population residing in locations with substantial heterogeneity in ambient UVR. Because of its large sample size, three more common ocular melanoma anatomical sites were able to be examined by sex, age, and race and ethnicity. Differences in the UVR effects by age, sex, and race and ethnicity may reflect differences in susceptibility in various populations. We found similar UVR effects across various age groups and by sex for total ocular melanoma and for the major anatomical sites. Differences in the UVR and ocular melanoma relationship by race and ethnicity may reflect differences in susceptibility in different populations. While we observed both positive and negative UVR associations across different races and ethnicities for various anatomical sites, tests of interaction were non-significant.

Misclassification of ambient UVR exposure may have resulted because average noontime ambient UVR was linked to location of residence only at diagnosis and individual exposure was not available. However, ambient UVR based on the Total Ozone Mapping Spectrometer database has been associated with individual UVR exposure measured by personal dosimeter [20].

In this cancer registry study, we do not have information on other potential risk factors for ocular melanoma that may induce bias into the study. Associations between UVR and ocular melanoma may reflect the differences in eye colour or skin pigmentation found in populations living at different latitudes. Unfortunately, we do not have information regarding geographical patterns in eye colour or skin pigmentation throughout the United States. Lifestyle factors, such as alcohol and tobacco use, may also potentially be associated with latitude. However, alcohol and tobacco use have not been strongly or consistently associated with ocular melanoma [4].

In summary, this U.S. cancer registry study does not support UVR as a risk factor of total ocular melanoma. By anatomical sites, we found UVR to be associated with an increased risk of ciliary body/iris melanoma, but a reduced risk of choroidal melanoma. Our results support and expand previous findings of associations of UVR and ocular melanoma and serve as a starting point for understanding the differences in the relationship between UVR and specific ocular melanoma sites.

## Summary

### What was known before

- Epidemiological studies on the relationship between solar UVR and risk of ocular melanoma have been inconsistent.
- Few studies reported risks for various anatomical sites of ocular melanoma or by race and ethnicity.

### What this study adds

- UVR was not a risk factor for all ocular melanoma sites combined.
- We found an inverse association between continuous UVR and total ocular melanoma (*N* = 18,089), largely driven by choroidal melanoma (*N* = 13,812), though a significant increased risk was found for ciliary body/iris melanoma (*N* = 2013).
- UVR was associated with increased risks of ciliary body/iris melanoma among non-Hispanic White individuals, but not among other races and ethnicities or for other anatomical sites.
- We found some evidence of an inverse association between this measure of ambient UVR and risk of choroidal melanoma among non-Hispanic White and Hispanic White individuals.

### Supplementary information

Supplementary tables 1–2

## Data Availability

All data used in this work is publicly available from the US Surveillance, Epidemiology, and End Results (SEER) Program of the National Cancer Institute. Specifically, this work used data from the database of Incidence – SEER Research Plus Limited-Field Data, 22 Registries, Nov 2021 Sub (2000–2019) - Linked To County Attributes - Total U.S., 1969-2020 Counties. These data can be downloaded using the software SEER*Stat, from https://seer.cancer.gov/seerstat/.
