# Supplementary material for: Ambient ultraviolet radiation and ocular melanoma incidence in the United States, 2000−2019
Source: Eye (Lond). 2024 Feb 13;38(9):1618–25. doi: 10.1038/s41433-024-02959-9 (PMC11156983; doi:10.1038/s41433-024-02959-9)
Supplement: Supplementary file 1 — Supplementary tables 1–2 [file 41433_2024_2959_MOESM1_ESM.docx]

| **Supplementary table 1**. Incidence rates across melanomas in six ocular anatomical sites diagnosed by age, sex, race and ethnicity, diagnosis year, and laterality in the Surveillance, Epidemiology, and End Results Program between 2000 and 2019 | | | | | | | | | | | | |
| --- | --- | --- | --- | --- | --- | --- | --- | --- | --- | --- | --- | --- |
|  | **Eye NOS** | | **Overlapping Region of Eye and Adnexa** | | **Orbit NOS** | | **Retina** |  | **Cornea** |  | **Lacrimal Gland** | |
|  | **Cases** | **IR (95% CI)** | **Cases** | **IR (95% CI)** | **Cases** | **IR (95% CI)** | **Cases** | **IR (95% CI)** | **Cases** | **IR (95% CI)** | **Cases** | **IR (95% CI)** |
| **All** | 622 | 0.21 (0.20, 0.23) | 208 | 0.07 (0.06, 0.08) | 181 | 0.06 (0.05, 0.07) | 104 | 0.04 (0.03, 0.04) | 45 | 0.02 (0.01, 0.02) | 25 | 0.01 (0.01, 0.01) |
| **Age (years)** |  |  |  |  |  |  |  |  |  |  |  |  |
| <50 | 99 | 0.05 (0.04, 0.06) | 33 | 0.02 (0.01, 0.02) | 36 | 0.02 (0.01, 0.02) | <25 | ^ | <25 | ^ | <25 | ^ |
| 50-64 | 206 | 0.40 (0.35, 0.46) | 62 | 0.12 (0.09, 0.15) | 56 | 0.11 (0.08, 0.14) | 39 | 0.08 (0.05, 0.1) | <25 | ^ | <25 | ^ |
| 65+ | 317 | 0.86 (0.77, 0.96) | 113 | 0.31 (0.25, 0.37) | 89 | 0.24 (0.19, 0.30) | 48 | 0.13 (0.1, 0.17) | 31 | 0.08 (0.06, 0.12) | <25 | ^ |
| **Sex** |  |  |  |  |  |  |  |  |  |  |  |  |
| Male | 314 | 0.22 (0.19, 0.24) | 101 | 0.07 (0.06, 0.08) | 103 | 0.07 (0.06, 0.09) | 49 | 0.03 (0.03, 0.04) | <25 | ^ | <25 | ^ |
| Female | 308 | 0.21 (0.18, 0.23) | 107 | 0.07 (0.06, 0.09) | 78 | 0.05 (0.04, 0.07) | 55 | 0.04 (0.03, 0.05) | <25 | ^ | <25 | ^ |
| **Ethnicity** |  |  |  |  |  |  |  |  |  |  |  |  |
| NHW | 570 | 0.34 (0.32, 0.37) | 198 | 0.12 (0.10, 0.14) | 155 | 0.09 (0.08, 0.11) | 93 | 0.06 (0.05, 0.07) | 38 | 0.02 (0.02, 0.03) | <25 | ^ |
| HW | 38 | 0.06 (0.04, 0.08) | <25 | ^ | <25 | ^ | <25 | ^ | <25 | ^ | <25 | ^ |
| Black | <25 | ^ | <25 | ^ | <25 | ^ | <25 | ^ | <25 | ^ | <25 | ^ |
| API | <25 | ^ | <25 | ^ | <25 | ^ | <25 | ^ | <25 | ^ | <25 | ^ |
| AI | <25 | ^ | <25 | ^ | <25 | ^ | <25 | ^ | <25 | ^ | <25 | ^ |
| **Year of Diagnosis** |  |  |  |  |  |  |  |  |  |  |  |  |
| 2000 - 03 | 149 | 0.27 (0.23, 0.32) | 74 | 0.14 (0.11, 0.17) | 40 | 0.07 (0.05, 0.10) | 31 | 0.06 (0.04, 0.08) | <25 | ^ | <25 | ^ |
| 2004 - 07 | 169 | 0.30 (0.26, 0.35) | 50 | 0.09 (0.07, 0.12) | 37 | 0.07 (0.05, 0.09) | 29 | 0.05 (0.03, 0.07) | <25 | ^ | <25 | ^ |
| 2008 - 11 | 136 | 0.23 (0.19, 0.27) | 33 | 0.06 (0.04, 0.08) | 37 | 0.06 (0.04, 0.09) | 27 | 0.05 (0.03, 0.07) | <25 | ^ | <25 | ^ |
| 2012 - 15 | 101 | 0.17 (0.14, 0.20) | <25 | ^ | 41 | 0.07 (0.05, 0.09) | <25 | ^ | <25 | ^ | <25 | ^ |
| 2016 - 19 | 67 | 0.11 (0.08, 0.14) | 27 | 0.04 (0.03, 0.06) | 26 | 0.04 (0.03, 0.06) | <25 | ^ | <25 | ^ | <25 | ^ |
| **Laterality** |  |  |  |  |  |  |  |  |  |  |  |  |
| Left-sided | 279 | 0.10 (0.08, 0.11) | 94 | 0.03 (0.03, 0.04) | 83 | 0.03 (0.02, 0.04) | 37 | 0.01 (0.01, 0.02) | <25 | ^ | <25 | ^ |
| Right-sided | 275 | 0.09 (0.08, 0.11) | 112 | 0.04 (0.03, 0.05) | 89 | 0.03 (0.02, 0.04) | 58 | 0.02 (0.02, 0.03) | <25 | ^ | <25 | ^ |
| Others^1^ | 68 | 0.02 (0.02, 0.03) | <25 | ^ | <25 | ^ | <25 | ^ | <25 | ^ | <25 | ^ |
| Abbreviations: IR, crude incidence rate (per 1,000,000 person-years); CI, confidence interval; NOS, not otherwise specified; NHW, on-Hispanic White; HW, Hispanic White; API, Asian /Pacific Islander; AI, American Indian.  ^, IR not calculated for fewer than 25 cases.  ^1^ Others included not a paired site; only one side or side unspecified; bilateral, single primary; paired site: midline tumor; paired site, but no information concerning laterality. | | | | | | | | | | | | |

| **Supplementary table 2**. Epidemiological studies investigating surrogates of solar UVR and ocular melanoma risk | | | |  |
| --- | --- | --- | --- | --- |
| **Study population** | **Number of cases** | **Anatomical sites** | **Main findings for solar UVR** | **Subgroup findings** |
| U.S. population-based cancer incidence among non-Hispanic White, Hispanic White, Black, Asian/Pacific Islander, and American Indian: SEER 22, 2000-2019 (current) | 18,089 | Sites specific: choroid, ciliary body/iris, conjunctiva, other sites of ocular melanoma combined | Null for overall ocular melanoma in higher modelled UVR exposure at diagnosis.  Reduced risk of choroidal melanoma in higher modelled UVR exposure at diagnosis.  Increased risks of ciliary body/iris melanoma, and other sites combined in higher modelled UVR exposure at diagnosis. | Similar results stratified by age at diagnosis, sex, and laterality.  Significant interaction by race/ethnicity (non-Hispanic White versus Hispanic White) and year of diagnosis; lower risk of choroidal melanoma appeared to be stronger in Hispanic White; high risk of ciliary body only remained in non-Hispanic White. |
| German case-control study with 3 different control groups (population-based, hospital-based, and sibling controls) (Schmidt-Pokrzywniak et al. 2009) | 459 | combined uveal: choroid, ciliary body, iris, and other uveal sites | Null for eye protection across 3 different controls.  Null for both worked outside >4 hours/day, and total lifetime occupational years of sun exposure versus two control groups; except versus hospital-based controls (increased risk for >15 years of occupational sun exposure). | Positive interaction reported for iris color and whether working outside >4 hours/day. |
| European population-based cancer incidence: EUROCARE, 1983-1994 (Virgili et al. 2007) | 5,566 | combined uveal: choroid, ciliary body/iris, and retina | Reduced risks in decreasing latitude (i.e., higher exposure to solar UVR) at diagnosis. |  |
| U.S. population-based cancer incidence among non-Hispanic White: SEER 12, 1992-2002 (Yu et al. 2006) | 2,142 | combined uveal (choroid, ciliary body and retina), and combined conjunctival melanoma (conjunctiva and cornea) | Reduced risk of combined uveal melanoma in decreasing latitude at diagnosis.  Borderline for increased risk of combined conjunctival in decreasing latitude at diagnosis (*P*=0.078). |  |
| Australian case-control study among White (Vajdic et al. 2002) | 290 | Site specific: combined choroid and ciliary body, conjunctiva, and iris | Choroid and ciliary body combined: Null in both latitude band at birth and at diagnosis.  Increased risks in longer total estimated occupational sun exposure hours. Null for total estimated recreational sun exposure hours.  Reduced risks in longer total lifetime modelled ambient UVR without a dose-response relationship. Null for eye protection.  Conjunctiva: null for all surrogates of UVR.  Iris: null for all surrogates of UVR. | Increased risks mainly in men; one additional increased risk of choroid and ciliary body combined in weekdays and weekends sun exposure combined in men. |
| French case-control study (Guénel et al. 2001) | 50 | combined uveal: choroid and ciliary body | Null for cumulative exposure to solar UVR, i.e., with exposure occurring in outdoor occupations based on job-exposure matrix. |  |
| Australian case-control study (Pane et al. 2000) | 125 | combined ocular: choroid, ciliary body, conjunctiva, and iris | Null for places and latitude.  Null for whether wearing prescription glasses and sunglass, but increased risk in about half the time of wearing sunglasses (versus less than half time), but null for the more than half time and all the time.  Null for cumulative ocular UVB exposure. | Reduced risks with increasing lifetime UVB exposure in fair-skinned subjects; but null in medium- and dark-complexioned subjects. Similar results stratified by intraocular melanoma (choroid, ciliary body, and iris), choroid and ciliary body combined, or choroid only. |
| U.S. case-control studies among White (Seddon et al. 1990)   Population-based series: | 197 | combined choroid and ciliary body | Reduced risk in southern place of birth (versus the North 40 degree).  Increased risk in longer cumulative years of residence in the North 40 degree (i.e., lower exposure to solar UVR).  Null for other surrogates of UVR. |  |
| Sibling series: | 337 | combined choroid and ciliary body | Increased risk of cumulative >40 years of intense sun exposure (versus 0 year).  Increased risk of rarely/never wearing sunglasses (versus almost always).  Reduced risk of high amount outside work (versus minimal).  Null for other surrogates of UVR. |  |
| U.S. case-control studies among White (Holly et al. 1990) | 407 | combined uveal: choroid, ciliary body, iris, and other uveal sites | Null for outdoor vacation and leisure outdoor time. | Increased risk of sunburn to eye or snow blindness, but this exposure variable combined with artificial UVR exposure including welding burn. |
| U.S. population-based cancer incidence: SEER 9, 1973-1984 studies among non-Hispanic White (Schwartz et al. 1988) | 763 | combined uveal: not specified | Null for birthplace.  Increased risk in southern birthplace (versus the north) in those who resided in a northern SEER area at the time of diagnosis.  Null for modelled ambient UVR. |  |
| Canadian case-control study among White (Gallagher et al., 1985) | 90 | combined ocular: not specified | Null for modelled ambient UVR.  Null for latitude and altitude of residence. |  |
| U.S. case-control studies among White with 2 types of hospital-based controls (Tucker et al. 1985)   Versus controls who had detached retinas | 444 | combined uveal: choroid, ciliary body and iris; some results showed iris specific | Increased risk of born in the south (versus born in the north).  Null for the number of years spent in the South.  Increased risk of occasionally or rarely eye protection in sun versus almost always. Null for eye protection in sun. Reduced risk in wearing glasses. Null for sunbathing, and outdoor leisure time. Increased risks of both outdoor hobbies, and more vacation sun exposure time. | Increased risk in less use of eye protection in sun in patients with iris melanoma only. |
| Versus controls who had ocular melanoma other than uveal melanoma | 444 | combined uveal: choroid, ciliary body and iris | Null for wearing glasses. |  |
| Abbreviations: UVR, ultraviolet radiation; EUROCARE, European cancer registry; SEER, Surveillance, Epidemiology, and End Results Program. | | | | |
